# Breeding Bread-Making Wheat Varieties for Organic Farming Systems: The Need to Target Productivity, Robustness, Resource Use Efficiency and Grain Quality Traits

**DOI:** 10.3390/foods12061209

**Published:** 2023-03-13

**Authors:** Leonidas Rempelos, Juan Wang, Enas Khalid Sufar, Mohammed Saleh Bady Almuayrifi, Daryl Knutt, Halima Leifert, Alice Leifert, Andrew Wilkinson, Peter Shotton, Gultekin Hasanaliyeva, Paul Bilsborrow, Steve Wilcockson, Nikolaos Volakakis, Emilia Markellou, Bingqiang Zhao, Stephen Jones, Per Ole Iversen, Carlo Leifert

**Affiliations:** 1 Lincoln Institute for Agri-Food Technology, University of Lincoln, Lincoln LN2 2LG, UK; 2 Nafferton Ecological Farming Group, Newcastle University, Newcastle upon Tyne NE1 7RU, UK; 3 School of Agriculture and Biology, Shanghai Jiao Tong University, Shanghai 200240, China; 4 Almadinah Regional Municipality, Medina 2020, Saudi Arabia; 5 Gilchester Organics, Stamfordham NE18 0QL, UK; 6 School of Animal, Rural and Environmental Sciences, Nottingham Trent University, Brackenhurst Campus, Nottinghamshire NG25 0QF, UK; 7 Geokomi Plc, Sivas Festos, 70200 Crete, Greece; 8 Benaki Phytopathological Institute, 14561 Athens, Greece; 9 Institute of Agricultural Resources and Regional Planning (IARRP), Chinese Academy of Agricultural Science (CAAS), Beijing 100081, China; 10 Bread Lab, Department of Crop and Soil Sciences, Washington State University, Burlington, WA 98233, USA; 11 Department of Nutrition, Institute of Basic Medical Sciences (IMB), University of Oslo, 0317 Oslo, Norway; 12 Department of Haematology, Oslo University Hospital, 0372 Oslo, Norway; 13 SCU Plant Science, Southern Cross University, Military Rd., Lismore 2480, Australia

**Keywords:** organic, conventional, crop breeding, selection methods, wheat, nutritional quality, baking quality

## Abstract

Agronomic protocols (rotation, tillage, fertilization and crop protection) commonly used in organic and conventional crop production differ significantly and there is evidence that modern varieties developed for conventional high-input farming systems do not have the combination of traits required for optimum performance in organic farming systems. Specifically, there is evidence that prohibition on the use of water-soluble, mineral N, P and K fertilizers and synthetic pesticide inputs in organic farming results in a need to revise both breeding and selection protocols. For organic production systems, the focus needs to be on the following: (i) traits prioritized by organic farmers such as high nutrient use efficiency from organic fertilizer inputs, competitiveness against weeds, and pest and disease resistance, (ii) processing quality parameters defined by millers and bakers and (iii) nutritional quality parameters demanded by organic consumers. In this article, we review evidence from variety trials and factorial field experiments that (i) studied to what extent there is a need for organic farming focused breeding programs, (ii) investigated which traits/trait combinations should be targeted in these breeding programs and/or (iii) compared the performance of modern varieties developed for the conventional sector with traditional/older varieties favored by organic farmers and/or new varieties developed in organic farming focused breeding programs. Our review focuses on wheat because there have been organic and/or low-input farming focused wheat breeding programs for more than 20 years in Europe, which has allowed the performance of varieties/genotypes from organic/low-input and conventional farming focused breeding programs to be compared.

## 1. Introduction

EU organic crop production standards prohibit or restrict the use of many external inputs that are widely used in conventional cereal production to ensure high grain yields and processing quality [1]. Specifically, organic farming standards prohibit the use of (a) mineral nitrogen (N), potassium chloride (KCl) and water-soluble phosphorus (P) fertilizers and (b) synthetic chemical crop protection products (including insecticides, acaricides, fungicides, herbicides, plant growth regulators and soil disinfection chemicals) [1]. 

Fertilization regimes in organic farming systems are largely based on regular inputs of organic fertilizers (e.g., manure and composts) and the use of legume crops in the rotation (to increase N levels and balance N:P ratios in the soil). However, restricted use of raw phosphate, potassium sulphate and mineral micronutrient fertilizers is permitted if shown to be necessary (by soil or plant analysis) to maintain soil fertility [1]. It is important to point out that EU environmental legislation also limits the amount of manure (the main N fertilizer available to organic farmers) that farmers can apply to their crops [2,3]. For example, in nitrate sensitive zones, the total N input from manure is limited to the equivalent of 170 kg N/ha/year, although inputs to specific crops can be higher (up to an equivalent of 250 kg N/ha) if this is balanced out by lower inputs to other crops in the rotation [2,3]. Both plant available and total N inputs from organic fertilizers to organic crops are therefore usually lower compared with the inputs of mineral N fertilizers to the same crops in conventional production systems in Europe [1,4,5,6,7,8,9].

It is also important to consider that N supply/availability profiles over the growing season differ considerably between mineral N and organic fertilizers such as animal and green manures [1,4,5,6,7,8,9,10]. The NH_4_^+^-N_,_ NO_3_^−^-N and urea-N in mineral N fertilizer products is immediately plant available and after application the concentration of plant available N fertilizer decreases steadily due to plant uptake, metabolism by soil micro-organisms and N losses (e.g., nitrate leaching, run-off or denitrification). In contrast, only a small proportion of total N in organic fertilizers is plant available NH_4_^+^ or NO_3_^−^, while a large proportion is present as organic N forms which only become available for plant uptake after mineralization in the soil [1,4,5,6,7,8,9,10]. Numerous studies have shown that only ~50% of N applied from green and animal manures becomes available to the crop planted immediately after application, although the amount of residual N that is available to subsequent crops is higher from manure when compared with mineral N fertilizer inputs [1,4,5,6,7,8,9,10]. N availability patterns from organic fertilizers are therefore less predictable and determined by parameters such as soil microbial activity and environmental parameters (soil organic matter content, temperature and soil matric potential) that affect mineralization processes [6,7,8,10,11]. 

The supply of plant available N is therefore thought to be the primary productivity limiting factor in organic crop production, especially in crop species which have a high N demand and/or low N uptake use efficiency (e.g., wheat and potato) [4,5,6,7,8,9,10,11,12]. There is mounting evidence that N availability not only affects plant growth and crop yield, but also the expression of resistance mechanisms and nutritional quality parameters in crops [1]. Specifically, phenolic compounds, which have anti-microbial activity, are a component of both constitutive and salicylic acid (SA)-inducible systemic resistance mechanisms in plants and it has been shown that increasing N availability to plants significantly reduces the concentrations of phenolic compounds and resistance against a range of biotrophic diseases in a dose-dependent manner [13,14,15,16,17]. Phenolics are also the main phytochemical group with antioxidant activity in crops whereby increased dietary intake of phenolics/antioxidants has been linked to human health benefits, including a reduced risk of cancer, type-2 diabetes and cardiovascular disease [1,18,19].

Crop health management in organic farming systems is based on preventative and non-chemical methods such as the use of (i) diverse crop rotations and regular organic green and animal manure inputs (to reduce weed competition and soil-borne pest and disease pressure), (ii) biological disease and pest control products and (iii) resistant/tolerant crop varieties/cultivars [1,20]. In addition, organic standards permit the use of biological control methods (e.g., the use of viruses, microbial antagonists and invertebrates/natural enemies for disease and pest control) and also certain mineral-based (e.g., Cu and S), plant extract-based (e.g., pyrethrum) and microbial extract-based (e.g., spinosad) crop protection products, when these treatments are used as a last resort [1,20,21]. 

There is also increasing evidence that non-use of mineral N fertilizer reduces the severity of a range of diseases (especially biotrophic fungal pathogens) and changes the profile of weed species in organic farming systems [1,20,22,23,24,25]. Similarly, there is mounting evidence that prohibition of the use of synthetic pesticides increases the density of microbial antagonists of soil-borne diseases and natural enemies (e.g., ground beetles) of invertebrate pests in agricultural ecosystems [1,20,26,27,28,29,30]. As a result, the profile and relative economic importance of different weed, disease and pest species can differ significantly between organic and conventional crop production systems [1,6,7,20,21,22,23,24,25,26].

Crop breeding programs focused on the needs of conventional farming systems have therefore been hypothesized to deliver varieties/cultivars that are unsuitable or lack essential traits needed in organic production systems [31,32,33,34]. 

In this article, we therefore review the currently available evidence that supports this hypothesis for cereals and in particular bread-making wheat. Specifically, we critically evaluate results from factorial field experiments which compared the performance of contrasting wheat varieties/genotypes (including comparisons of varieties from organic and conventional farming focused breeding/selection programs) in the context of contrasting crop rotation, fertilization and/or protection regimes used in organic and conventional production systems. 

Consumer expectation that organic food crops have a superior nutritional composition and sensory quality is an important driver for the increasing demand for organic foods and organic farmers [1,21,35]. This review therefore also focuses on studies that allowed trade-offs and/or synergies between (i) crop performance (crop health, growth and/or yield parameters) and (ii) nutritional, processing and/or sensory quality traits to be assessed in varieties from contrasting breeding programs and different agronomic backgrounds. 

## 2. Agronomic Protocols Used in Organic and Conventional Wheat Production

The agronomic protocols used for organic wheat production in Northern and Southern Europe differ substantially from those used in intensive conventional production in terms of (i) tillage, (ii) rotational design, (iii) fertilization regimes and (iv) crop protection practices [4,6,7,9,36,37,38]. 

### 2.1. Tillage

Reduced tillage is now widely used in conventional arable farming systems, while many organic farmers continue moldboard ploughing before planting of wheat crops primarily to (i) incorporate green and animal manures, (ii) control weeds [39,40,41,42] and (iii) reduce disease inoculum through the burial of trash and stubble. Mechanical weed control based on tine weeders and inter-row weeding systems is also more widely and frequently used in organic production systems, with herbicides being the main method of weed management in conventional production systems [1,4,6,7,9,23,43,44,45]. 

Long-term field experiments in both Northern Europe and North America suggest that, overall, the efficacy of mechanical weed control protocols used in organic farming is lower than the herbicide-based protocols used in conventional farming [23,42,43]. Both the lower efficacy and crop damage associated with mechanical weed control protocols may have significant negative effects on wheat yields and quality in organic farming [45]. 

### 2.2. Rotational Design

Conventional wheat production is primarily based on stockless, short, cereal-dominated rotations and only farms which continue to have livestock regularly include pure grass or mixed grass–legume leys in the rotation [1,46,47]. In addition, many conventional arable rotations include (i) only one break crop (e.g., oilseed rape in many regions of Northern Europe) and (ii) second wheat crops (wheat grown after wheat), especially when wheat prices are high. In addition, when maize is included in the rotation, wheat may be established after maize crops in conventional arable rotations, although it increases the risk of *Fusarium* disease and mycotoxin contamination in the harvested grain [1,46,47].

In contrast, a large proportion of organic wheat is produced in mixed farming systems which usually have a 2–3-year pure legume or mixed grass/legume sward in the rotation [1,46,47]. Where wheat is produced on stockless organic farms, rotations usually include a 2–4-year legume or grass/legume ley phase for fertility building. In organic systems, wheat is often grown immediately after fertility building leys to achieve higher yields and/or to achieve the minimum bread-making quality standards (e.g., protein concentrations of 13% in the UK) set by processors for premium prices [4,7,37]. Overall, organic rotations in Europe tend to be more diverse and may include field vegetables, potatoes and grain legumes; wheat is rarely grown following wheat or maize in the rotation (due to increasing the risk of pest and disease damage) and oilseed rape is less frequently used when compared with conventional arable rotations ([1,7,46,47]). 

### 2.3. Fertilization Regimes

In the main wheat growing region of Northern Europe, conventional common wheat crops (*Triticum aestivum* L.) receive mineral N fertilizer inputs often in excess of 200 kg N/ha, although lower N inputs (~100 kg/ha) are usually applied to spelt wheat (*Triticum spelta* L.) [4,9,36,37,38]. Substantial P (up to 110 kg P_2_O_5_/ha) and K (up to 150 kg/ha K_2_O) inputs are also applied to wheat crops, but input levels vary widely depending on the residual soil P and K levels; many conventional farmers use soil analysis and management information (e.g., soil type and previous crop and straw incorporation or removal) for decision making on P and K inputs [36]. 

It is more difficult to estimate the mean total and available N inputs to organic wheat crops, since (i) residual N input from preceding legume leys is both highly variable and difficult to measure and (ii) both total and available N in animal manure can vary greatly depending on manure type, processing and storage methods [1,9,36,37]. However, because (a) only a proportion (often <50%) of total N from both green and animal manure is considered to be available to the first crop planted after manure inputs and (b) because environmental legislation limits input of animal manure to 170 kg N/ha, the amount of N available to organic cereal crops is significantly lower when compared to mineral N inputs in conventional systems. While mineral N fertilizers are prohibited, organic farmers are able to supplement P and K inputs with permitted mineral P (finely ground rock phosphate) and K (K_2_SO_4_) fertilizers [1]. As a result, nitrogen is thought to be the primary growth and yield limiting nutrient in most organic production systems [48]. 

### 2.4. Crop Protection

Conventional crop protection protocols rely on the intensive use of synthetic chemical crop protection inputs. For example, according to the UK Pesticide Usage Survey in 2016, UK winter wheat crops on average received 3.6 fungicide, 2 plant growth regulator and 3 herbicide treatments, plus 1 insecticide treatment (https://secure.fera.defra.gov.uk/pusstats/surveys/index.cfm; accessed 26 February 2023). However, the combinations and amounts of pesticides differ considerably throughout Europe, depending on (a) climatic conditions, (b) regional pest, disease and weed pressure and (c) levels of restriction from national environmental legislation [49,50]. 

In contrast, the use of synthetic chemical pesticides is prohibited in organic farming systems and crop protection is based on cultural and mechanical control, although some organic farmers use (i) plant extract (e.g., tillekur) based fungicides as seed treatments for the control of seed-borne diseases, (ii) sulfur fungicides for foliar disease control and/or (iii) plant extracts (e.g., pyrethrum) and/or microbial fermentation (e.g., Spinosad) based insecticides for pest control in cereals [1]. 

Results from long-term, factorial field experiments have demonstrated that the severity and ranking of crop protection challenges, in terms of economic impact and relative need for intervention, differ considerably between conventional and organic wheat production systems. For example, in Northern Europe the severity of lodging and biotrophic diseases such as mildew and rust in manure-fertilized organic crops was (i) significantly lower than in mineral NPK-fertilized crops grown without fungicide/growth regulator treatment and (ii) below the threshold at which fungicide/growth regulator applications would become economically viable in conventional farming [4,6,7]. In addition, a recent literature review by Bernhoft et al. [51] concluded that, overall, the risk of *Fusarium* head blight and mycotoxin contamination of wheat grain is lower in organic compared with conventional production systems. They describe a range of agronomic factors linked to an increased risk of *Fusarium* infection and mycotoxin levels in conventional production. Interestingly, risk factors in conventional systems include (i) minimum tillage, (ii) short rotations, especially growing wheat after wheat or maize, (iii) high N fertilizer inputs, and (iv) the use of certain types of fungicide (e.g., strobilurins) and the growth regulator chlormequat, which is used to reduce stem length and the risk of lodging in wheat [51]. 

In contrast, when leaf blotch (caused by *Septoria tritici*) severity was compared in a modern UK short straw variety (Malacca), disease severity was similar in both organic and mineral NPK-fertilized crops grown without fungicide/growth regulator treatment [4]. Disease severity in both systems was above the level at which fungicide treatments are economically viable in conventional cereal production [4]. 

## 3. Wheat Breeding/Selection Objectives

### 3.1. Productivity

Meta-analyses of comparative cereal yield data found that grain yields in organic cereal production are significantly (15–30%) lower than those achieved in intensive, conventional production [48,52,53,54,55]. These estimates were confirmed by results from long-term, factorial field experiments with common wheat (*T. aestivum*) in Northern Europe and North America, which also demonstrated that differences in both crop protection protocols and fertilization regimes contribute to the yield gap [4,6,7,43] (see Table 1 as an example). 

In organic production systems, weed competition and foliar disease caused by *Septoria* spp. were identified as the major yield-limiting crop protection challenges that may be addressed by crop breeding/selection [1,6,7,31,32]. In addition, bunt resistance has been identified as an important breeding target, especially for those organic farming systems which regularly save their own seed for planting in the next growing season [31,32,56]. 

Recent factorial field experiments with both common and spelt winter wheat in the UK demonstrated that grain and protein yields and important yield-influencing factors (e.g., *Septoria* and lodging severity) are determined by complex interactions between (a) agronomic practices used in organic and conventional wheat production, (b) variety and (c) pedo-climatic background conditions [1,6]. For example, a recent study in the UK detected significant interactions between fertilizer type, fertilizer input level, and crop protection and/or variety for (a) grain and protein yields and (b) both stem lodging and *Septoria* severity, which are major grain yield and quality influencing parameters in the UK (Table 1).

Lodging was detected in the longer straw variety Aszita (a variety developed for the organic farming sector) but not the modern short straw variety Solstice [6]. For Aszita, a significant interaction between fertilizer type and crop protection was detected for lodging severity, which was low and not significantly affected by crop protection when cattle manure was used as a fertilizer (Table 2). In contrast, in mineral N fertilized Aszita crops, lodging was more than two times higher when organic crop protection regimes were used (Table 2). This was most likely due to the substantially higher concentrations of readily plant available forms of nitrogen (NH_4_-N and NO_3−_N) being available in crops fertilized with mineral N compared with cattle manure, since the risk of lodging was previously reported to increase with increasing the mineral N fertilizer inputs in wheat [1,6,37].

Azita and Solstice produced the same grain yield with cattle manure as the fertilizer, while Solstice produced significantly higher grain yields than Aszita with mineral N fertilizer (Table 3). 

*Septoria* severity on flag leaves was significantly higher in Solstice than Aszita (Table 1). However, while *Septoria* severity in Aszita was not significantly different in crops under organic and conventional crop protection, conventional crop protection significantly reduced *Septoria* severity in Solstice (Table 4).

Significant three-way interactions between fertilizer type, fertilizer input level and variety were detected for *Septoria* severity on the second leaf (L2) and grain protein concentrations (Table 1). 

When these interactions were further investigated, *Septoria* severity was similar in Aszita and Solstice when cattle manure at the higher input level (170 kg N/ha) was used as the fertilizer. In contrast, *Septoria* severity was significantly higher in Solstice with the other three fertilization regimes (Table 5).

Grain protein concentrations were significantly higher in Aszita compared with Solstice with all four fertilization regimes (Table 5). In addition, protein concentrations were highest with mineral N applied at the higher input level (170 kg N/ha). However, the relative differences in protein concentration between the four fertilization regimes were greater with Aszita (Table 5).

Differences in N uptake efficiency/N harvest index between the two varieties are likely to at least partially explain these results, but the exact physiological mechanisms underlying these interactions have not been investigated. However, investigations into the physiological and genetic mechanisms responsible for these interactions may lead to the discovery of new breeding strategies for (i) grain and protein yield/yield stability in organic systems and (ii) resistance against *Septoria*, the main yield affecting disease in both organic and conventional production [6]. 

Overall, the currently available evidence suggests that lodging, foliar diseases caused by biotrophic fungi such as powdery mildew and rusts, and plant residue/soil-borne diseases such as *Fusarium* head blight and take-all (which can cause major yield losses in intensive conventional systems) have no or a lower impact on grain and protein yields in organic compared with conventional wheat production [1,6,7,51].

More recently, a modelling-based study by Döring and Neuhoff [48] concluded that the inability to increase N availability in organic farming systems via biological nitrogen fixation from legume crops is the main barrier for closing the organic/conventional yield gap for wheat in Northern Europe. 

This view is supported by results from long-term field experiments which compared the performance of modern, short straw common wheat varieties in organic and conventional management systems [4,6,7,9]. These studies reported not only lower grain yields, but also lower protein concentrations and/or chlorophyll levels (traits that are known to be positively correlated with N availability/supply) in organically grown crops [4,6,7,9]. However, recent trials carried out in northern Britain reported that winter wheat varieties from organic wheat breeding programs in Switzerland produced similar yields and higher protein levels in organic production when compared with intensive conventional winter wheat production systems in the region [57].

Multi-site variety trials with six contrasting spring wheat varieties carried out on organic farms located in three contrasting pedo-climatic zones of the UK also identified very highly significant (*p* < 0.001) interactions between site/environment and variety for (i) grain and protein yield, (ii) major yield-determining parameters such as *Septoria* and yellow rust severity and (iii) processing and nutritional quality parameters [57] (Table 6, Table 7 and Table 8).

Although no varieties specifically developed for organic farming systems were included in these trials, the study demonstrates the need to (i) focus on high levels of disease resistance and (ii) use local/regional selection/adaptation strategies in breeding programs for the organic sector as previously described [31,32,33]. Correlation analyses of data from these trials showed (i) that both leaf chlorophyll and grain yield were negatively correlated with grain protein content and (ii) a weak positive correlation between stem length and protein content (Supplementary Table S1). These results were consistent with previous studies that reported (i) negative correlations between grain yield and protein content and (ii) positive correlations between stem length and protein content and/or bread-making quality of wheat grain [1,6,32,33,37,38,57]. See supplementary materials for a more detailed description and discussion of results from the correlation analyses. 

Overall, trials with both winter and spring wheat varieties of common wheat (*T. aestivum* L.) identified that the yield gap between organic and conventional production may be significantly reduced with organic farming focused common-wheat breeding and selection protocols.

The importance of considering environment x variety interactions and local selection/adaptation when developing varieties for the organic sector is also highlighted by recent factorial field experiments with four contrasting winter spelt wheat (*T. spelta*) varieties in (a) rain-fed production systems in Northern Europe (UK) and Central Europe (Czech Republic) and (b) both rain-fed and irrigated wheat production in a semi-arid region in Southern Europe (Crete, Greece) [9,37].

In the UK, a traditional long straw spelt variety (Oberkulmer) produced significantly higher grain yields than the varieties Filderstolz, Rubiota and Züricher Oberländer Rotkorn (ZOR). In contrast, Filderstolz, a short straw variety developed from a *T. aestivum* × *T. spelta* cross for the conventional farming sector, produced the highest grain yields in the Czech Republic [9], and ZOR, a variety developed for the organic farming sector, produced the highest yields under semi-arid conditions in Crete [37]. The variety trials in Crete also identified a significant interaction between variety and irrigation (with and without supplementary irrigation). Although ZOR produced numerically higher yields than all other varieties in both rain-fed and irrigated systems, significant yield differences between varieties were only detected in irrigated crops.

It is important to note that significant main effects of fertilizer type and input level were also identified in all three countries. Specifically, the highest yields were produced by (i) digestate from a commercial biogas fermenter unit using energy crops as the only feedstock produced the highest grain yields in the UK, (ii) mineral fertilizer in the Czech Republic and (iii) sheep and chicken manure in Greece. In these trials, mineral N and organic fertilizers were applied at the same total N input level of 100 kg N/ha. However, although digestate and manure-fertilized crops produced significantly higher yields than mineral N fertilized crops in both the UK and Greece, grain protein contents were highest in mineral N fertilized crops in all three countries [9,37]. Significant interactions between fertilizer type and spelt variety were only detected in the Czech Republic [9]. 

When considering the relative importance of grain yield as a breeding target for the organic sector, it is important to take into account that a long-term farming system comparison in the Netherlands found that the organic–conventional yield gap narrows over time, and that this may be due to the development of higher nutrient use efficiency and “spatial stability” in the organic system over time [54]. 

The most important yield-related breeding objectives suggested for organic farming focused wheat breeding/selection programs were (i) nutrient use efficiency (in particular N uptake efficiency from organic fertilizers), (ii) competitiveness against weeds, (iii) capacity to recover from mechanical weed control damage and (iv) *Septoria* and bunt resistance/tolerance [1,6,7,31,32,56,58,59].

### 3.2. Protein Concentrations and Processing Quality Traits

Most retail and farm surveys and long-term field experimental studies that compared protein and other processing quality parameters in organic and conventional farming have reported lower concentrations of protein in organic wheat grain or flour/products comparators [4,6,7,9] (see Table 1, Table 3 and Table 8 for examples). However, one long-term field experiment (the DOC trial in Switzerland) reported no significant differences in protein concentrations and baking quality between organic and conventional wheat grain over a 21-year observation period, although it should be pointed out that the same grass/clover ley–cereal–field vegetable rotations (typical for mixed farming systems)and similar total N input levels were used in the organic and conventional systems [60]. 

The relative demand for wheat grain for use as animal feed is thought to be lower in the organic sector, because (i) organic farming standards prescribe high levels of outdoor grazing/scavenging and severely restrict the proportion of concentrate feeds in ruminant diets and also restrict the use of imported concentrate feeds for pig and poultry production and (ii) because relative consumption of animal products (especially meat) was reported to be lower in cohorts with high organic food consumption levels when compared to cohorts consuming exclusively conventional foods [1,61,62]. 

In contrast, there is a higher demand for wholegrain cereal products and in particular wheat grain that has high bread-making quality from wholegrain and stone-ground flour [63] and wheat varieties suitable for long fermentation (sour dough-type) breadmaking processes [64]. The higher demand is thought to result from greater awareness among organic consumers about the nutritional/health benefits linked to wholegrain products and sourdough bread consumption [61,62,63,64,65]. 

The lower protein levels achieved by modern varieties in organic production systems are thought to be one of the main reasons why organic crops (and especially winter wheat crops) more frequently fail to achieve the threshold protein levels demanded by processors for baking quality price premiums, although thresholds are often lower than for conventional crops [4,6,7] (see results obtained for the variety Solstice in Table 1 and Table 3 as an example). It also, at least partially, explains why (i) a significant proportion of organic producers have continued to use traditional, older, longer-straw varieties with lower maximum yield potential, but with the potential to produce high grain protein concentrations and bread-making quality [4,6,7,66] and (ii) spring wheat crops, which are known to more reliably achieve high protein levels than winter wheat crops in many Northern European regions (see Table 6, Table 7 and Table 8 as an example), are preferred by organic farmers in many Northern European regions [57,59]. However, achieving satisfactory baking/bread-making quality with modern spring wheat varieties in conventional farming systems also remains a challenge, especially in Northern Europe [59,67].

Increasing protein content and other processing quality-related traits has therefore been one of the most important objectives in organic farming focused breeding/selection programs for bread-wheat in Europe [31,32,33,34]. 

### 3.3. Nutritional Quality Traits

Systematic literature reviews/meta-analyses, long-term factorial field experiments, retail surveys and dietary intervention studies reported that organically produced wheat products contain (i) higher levels of nutritionally desirable minerals, phenolics and/or antioxidant activity and/or (ii) lower concentrations of nutritionally undesirable/toxic pesticides, Cd and/or *Fusarium* mycotoxins [1,21,35,38,68,69,70,71,72,73]. In addition, a recently published retail survey in the UK and Germany demonstrated that relative differences in nutritional composition between organic and conventional wheat flour were significantly greater when wholegrain instead of refined wheat flour was compared [71,72,73]. The mounting evidence for higher nutritional quality of organic foods and potential positive health impacts from organic food consumption is therefore likely to (a) reinforce existing consumer perceptions that organic cereal products have a higher nutritional value and (b) increase demand for organic and especially organic–wholegrain cereal products [1,21]. 

Factorial field experiments in both Northern and Southern Europe reported significant effects of variety and fertilizer type (mineral N or NPK versus manure) on a range of nutritional quality parameters including antioxidant capacity and concentrations of phenolics, mineral micronutrients (e.g., Ca, Fe and Zn) and the toxic metal Cd [6,57,74].

Experiments with common winter wheat in the UK reported higher levels of phenolics and mineral micronutrients when manure and a longer straw variety (Aszita) developed for the organic sector were used compared with mineral N as the fertilizer and the modern short straw variety Solstice, respectively [6,57]. The trials in Northern Europe also identified significant interactions between variety and fertilizer type on leaf phenolic and flavonoid and Cd concentration (Table 9). Specifically, the relative difference between varieties was greater when cattle manure rather than mineral N was used as the fertilizer (Table 9). There was no significant difference in grain Cd concentrations between varieties when fertilized with manure, while Cd concentrations were significantly higher in the variety Aszita when fertilized with mineral N (Table 9). 

Experiments with common spring wheat varieties in the UK also identified very highly significant (*p* < 0.001) main effects of variety on grain Cd and mineral micronutrient (Ca, Fe, Zn) concentrations, but did not compare phenolic levels in leaves or grain [57].

In addition, these experiments identified highly significant environment x variety interactions (trials were carried out in two contrasting seasons and in three UK sites with contrasting pedo-climatic conditions) for a range of nutritional quality parameters [57]. 

Correlation analyses of data from spring wheat variety trials carried out on organic farms in three contrasting pedo-climatic environments in the UK identified negative correlations between grain yield and concentrations of Cd, Fe and Zn. This suggests that the use of varieties with a high yield potential and/or breeding/selection for a high grain yield in organic farming may have both positive (lower grain Cd concentrations) and negative (lower Fe and Zn concentrations) impacts on nutritional quality parameters (Supplementary Table S1). See supplementary materials for a more detailed description and discussion of results from the correlation analyses. 

Trials carried out with winter spelt wheat in a semi-arid region (Crete, Greece) reported that (i) the highest phenolic concentrations and total antioxidant capacity were found in grain from the short straw variety (Filderstolz) developed for the conventional sector, while (ii) the highest Fe and Zn concentrations were found in grain from the traditional, long straw Swiss variety (Oberkulmer) [38]. 

Nutritional quality related traits, although rarely considered/targeted in conventional wheat breeding programs, are expected to be of increasing importance for organic farming focused wheat breeding/selection programs, especially if nutritional/health benefits linked to older, traditional wheat species and/or varieties are confirmed [1,75,76].

Breeding for nutritional quality traits is likely to be linked to selection for baking and bread-making quality from wholegrain flour, because (i) refining removes most of the nutritionally desirable mineral micronutrients, phenolics, vitamins and antioxidants from wheat grains/flour and (ii) the relative demand for wholegrain products is thought to be greater among organic consumers [1,61,62].

## 4. Breeding/Selection Methods, Strategies and Approaches

A range of strategies have been used to develop wheat varieties/cultivars and/or cultivar mixtures suitable for the needs of the organic sector and these are described below. 

### 4.1. Traditional Breeding Programs

The first systematic breeding programs for organic farming systems were developed by Peter Kunze (a biodynamic wheat breeder) in collaboration with Sativa Biosaatgut GmbH (www.sativa-biosaatgut.de (accessed 26 February 2023) and focused on developing bread-making winter wheat varieties. They were based on (i) targeted crosses of modern high-performance wheat cultivars and older longer-straw varieties/landraces from Germany and Switzerland and (ii) subsequent farmer participatory selection in commercial organic farming backgrounds with different pedo-climatic environments [6,57,77]. This approach led to the development of commercially successful organic varieties and similar breeding/selection approaches are now utilized for both spring and winter common wheat (*T. aestivum*) and more recently also durum wheat in Europe [78,79,80,81].

As described above, factorial field experiments in the UK demonstrated that the longer-straw, winter wheat variety Aszita (a Sativa variety developed via an organic farming focused breeding program) had similar grain yields, but (i) significantly lower foliar disease levels of *Septoria* and rust and (ii) higher leaf phenolic concentrations, protein contents and bread-making quality when grown in organic agronomic background conditions when compared to the modern short straw Solstice (which was developed via a conventional farming focused breeding program) [6]. Aszita had lower lodging resistance than Solstice, but, as in previous long-term, factorial field trials with the conventional variety Malacca [4,7], significant levels of lodging were only observed when mineral N fertilizer was used without protection by synthetic chemical fungicides and plant growth regulators [6]. Superior performance under organic farming conditions has been confirmed for Aszita and other Sativa organic bread-making wheat varieties in variety comparison trials in the UK and other locations in Northern Europe [57,77,82]. 

Based on the success of these pioneering organic breeding programs, it is now widely accepted that selection of varieties for the organic farming sector should be carried out in agronomic backgrounds that reflect crop rotation, mechanical weed control practices and fertilization regimes used in commercial organic farming practices. 

### 4.2. Molecular Breeding Tools/Strategies 

Traditional wheat-breeding programs, which are based on crossing parental lines and subsequent selfing of the offspring for several generations to obtain inbred lines, usually takes a minimum of 10 years to bring a variety to market. In addition, many of the functional traits desired by organic and conventional farmers have low heritability and/or are difficult or expensive to select using phenotypic selection methods resulting in slow genetic gains. To speed up/improve breeding progress, a range of studies have investigated the potential for Marker Assisted Selection (MAS) for wheat improvement [83,84,85]. 

Although most of these studies have focused primarily on breeding priorities for the conventional sector (e.g., grain yield, *Fusarium*, powdery mildew and rust resistance), some have targeted important functional traits desired by organic farmers (e.g., nutrient-use efficiency, bunt and *Septoria* resistance, bread-making quality, etc.) [56,86,87]. This included the EU project NUE crops which investigated gene and protein expression patterns in wheat varieties developed for the organic and conventional farming sector grown with contrasting fertilizer input types (manure versus mineral N) and input levels in order to identify quantitative trait loci (QTLs) that may be used as molecular markers for N use efficiency traits [11,88,89].

While these molecular breeding tools are increasingly used to develop new wheat varieties for the conventional, high-input farming sector, it is difficult to assess (i) to what extent they have been used in organic farming focused breeding programs and/or (ii) whether they have improved or speeded up conventional farming focused breeding programs, since such information is usually kept confidential by seed companies. 

In this context, it is important to consider that grain yields (the primary breeding target in conventional farming) have stagnated in many regions of Europe for the last 20 years, although the yield potential (yield achieved with optimized mineral NPK fertilizer input and crop protection regimes) of wheat varieties has continued to increase [1,90,91]. This has been attributed to a range of factors, including (i) rising mineral fertilizer and crop protection product costs, (ii) environmental legislation limiting fertilizer inputs and/or banning/restricting the use of certain crop protection products, (iii) climate change and (iv) increasing resistance to applied pesticides [92,93,94,95]. 

### 4.3. Farmer Participatory Breeding Approaches

Farmer participatory breeding has been used widely in developing countries which have limited resources for the development and in particular field evaluation/regional adaptation of new varieties [96]. However, the benefits of participatory plant breeding (PPB) approaches are now also increasingly recognized by wheat breeders in developed countries, especially during the final stages of selection [33,96,97,98].

PPB approaches have been widely adopted in organic farming focused breeding programs, not only because they can reduce the cost, but also because they increase the efficiency of the final selection for farm or region-specific quality-related traits, functional traits and/or trait combinations for diverse production environments [32,33]. 

Adaptation of wheat varieties to local or regional pedo-climatic and also agronomic background conditions is widely considered to be particularly important for the organic farming sector [32,57,67]. This is mainly because most agrochemical interventions, which generate a “level playing field” across environments in conventional farming (with respect to both soil fertility andweed, disease and pest pressure), are prohibited in organic farming [1]. In addition, adaptation is necessary because agronomic protocols used in organic farming are more diverse, which in turn is due to the need to adjust rotations, fertilization and crop protection regimes to (i) local pedo-climatic conditions, (ii) pest, disease and weed pressures, and (iii) farm-specific input availability (e.g., type of manure or organic waste based composts) [32,57,67].

This view is supported by factorial field experiments that investigated environment × agronomy × variety (E × A × G) interactions [9,57,79]. For example, when the performance of six contrasting common spring wheat varieties was compared on three UK organic farms with contrasting pedo-climatic background conditions, a range of complex interactions were identified and the ranking of varieties differed considerably for both yield and quality parameters (Table 6, Table 7 and Table 8). In addition, when the performance of the variety Paragon grown with different organic fertilizer input types (chicken manure pellets, cattle farmyard manure and green waste compost) and fertilizer input levels on the same three farms were analyzed, a range of complex A × E interactions were detected [57]. 

The need for farmer participation throughout the breeding program (from the selection of parents for crosses to local/regional selection/adaptation) is also increasingly recognized and advocated [67,97].

### 4.4. Evolutionary Plant Breeding

Evolutionary plant breeding (EPB) has been advocated as an approach to develop locally adapted wheat cultivars, usually described as “heterogeneous populations”, for organic farming systems [99]. EPB is based on a cycle of sowing mixtures of varieties or crosses of different varieties with a high level of genetic diversity and then resowing seeds year after year and relying on natural selection for the development locally adapted evolving populations (EP). EP are now available for commercial wheat production in some European countries and the philosophy, methods, relative performance of wheat populations and challenges of EPB have been reviewed extensively [32,67,99,100,101,102]. For example, a recent assessment of EP performance in the US reported that “*EPs performance was dependent on their pedigree and were statistically similar and even out-performed some of their respective parents in regards to grain yield, grain protein concentration, and disease resistance*” and that both “*bi-parental and composite-cross populations demonstrated significantly greater stability over the parents across precipitation zones, confirming the capacity of genetically diverse EP populations to adapt to different environments*” [102]. In particular, reduction in disease incidence/severity and pest pressure in crops established from wheat populations is now well documented [103,104,105,106,107,108,109,110] and exploited in commercial practice by organic producers and increasingly also by conventional farmers that follow ‘low-input’ and/or regenerative farming protocols. For example, it has been reported that in France the area of bread-making wheat varieties grown in mixtures has increased from 5% in 2015 to 12% in 2020 [107]. In the UK, grain from wheat populations can be marketed for use as animal feed and the production of distillery products, but some on-farm bakeries sell bread made with flour from a mixture of contrasting wheat crosses [110]. In addition, the UK Agricultural and Horticultural Development Board now provides a “Variety blend tool for winter wheat” for farmers on its website [107].

### 4.5. Breeding for Nutritional Quality Traits

Recent factorial field experiments [6,38] suggest that both (i) the use of organic instead of mineral N fertilizers and (ii) differences in variety choice contributed to higher concentrations of phenolics and/or antioxidant activity reported in organic wheat products [35,71]. In contrast, higher concentrations of mineral micronutrients in organic wheat appear to be primarily linked to differences in variety and specifically the more widespread use of older, traditional and/or longer-straw varieties in organic farming systems [6,38,66]. However, it should be pointed out that higher Cu concentrations in organic compared with conventional wheat grain were also reported to result from the use of Cu-fungicides in crops grown before wheat (e.g., for blight control in organic potato crops) [111].

Higher concentrations of the toxic metal Cd in conventional wheat grain were linked to higher inputs of mineral P fertilizers (which contain Cd) [111], but recent variety trials with spring and wheat also identified significant main effects of variety [57,112] (see Table 7 for an example). 

To our knowledge, parent lines used for crosses and progeny generated in organic breeding programs are not currently tested/selected for nutritional quality parameters other than protein content. Higher phenolic and mineral micronutrient concentrations in organic wheat grain are therefore thought to have been due to (i) the contrasting variety choices made by organic farmers and/or (ii) correlations between nutritional composition parameters and other functional traits that were used for selection of parents and/or progeny in organic farming focused breeding programs. 

Evidence supporting this hypothesis comes from (i) variety trials which compared the performance of modern wheat varieties developed for the conventional farming sector with older, longer-straw varieties that continue to be used by a significant proportion of organic wheat producers [66] and (ii) factorial field experiments which compared the performance of wheat varieties from organic and conventional farming focused breeding programs in contrasting agronomic backgrounds [6]. For example, organic farmers preferring organic breeding programs tend to select for longer-straw phenotypes to improve competitiveness against weeds and protein content/bread-making quality (parameters which are thought to be positively correlated with straw length) and there is increasing evidence that longer-straw varieties of both common and spelt wheat also produce grain with higher mineral micronutrient concentrations [1,6,38] (see Table 1, Table 2, Table 3, Table 4, Table 8 and Table 9 for examples). 

There is also some evidence that selection for longer-straw varieties may co-select for improved capacity for mineral micronutrient acquisition and/or translocation into the grain. Specifically, a recent study into the evolution of mycorrhizal competence and grain traits in wheat and other cereals reported that (i) the introduction of semi-dwarfing genes to shorten stem length has reduced the capacity of major cereal species to develop and gain the full benefits (which include micronutrient uptake) from mycorrhizal associations [113] and (ii) both straw length and the mineral content of wheat grain has gradually decreased over time since the start of the introduction of semi-dwarfing genes in the early 1970s [114].

In this context, the complex interactions between crop protection, fertilization and variety identified in factorial field experiments (see Section 3 for examples) could be of particular interest for the design of future nutritional quality focused breeding programs for both common and spelt wheat. For example, when compared to the short straw, conventional variety Solstice, grain Zn concentrations were significantly higher in grain from the long straw, organic variety Aszita with both manure and mineral N fertilizer, while grain concentrations of the toxic metal Cd were significantly higher in Aszita only when mineral N fertilizer was used (Table 3). In addition, longer straw varieties of Spelt wheat developed for or preferred by organic farmers had higher grain Fe and Zn, but not Cd concentrations when compared to the modern short straw spelt variety (Filderstolz) developed for the conventional sector (Table 8). This suggests that the physiological parameters responsible for mineral micronutrients in grain differ from those that determine Cd concentrations and that the use of organic varieties in conventional production systems may have negative trade-offs (e.g., higher grain Cd levels) with respect to nutritional quality. This conclusion is supported by (i) the correlation analyses of data from organic spring wheat variety trials (see supplementary material) and (ii) a recent genome-wide association study (GWAS) in wheat which identified five new cadmium uptake loci and evidence that there are no positive correlations between the uptake of Cd and mineral micronutrients such as Fe and Zn in common wheat [115]. 

It is important to point out that significantly lower Cd concentrations in Solstice grain (compared with Aszita) were only detected in mineral N fertilized crops not protected with fungicides and chlormequat (Table 4). Similar results were also reported in a recent study by Motta-Romero et al. [112] who studied the effects of foliar fungicide applications on yield, micronutrients and cadmium in grains from historical and modern hard wheat genotypes. When they compared varieties developed in different time periods in two field experiments, grain Cd significantly increased over time (0.4 μg/kg/yr; *p* < 0.01) only when varieties were grown without protection from fungicides. It is therefore feasible that differences in grain Cd (and possibly also mineral micronutrient) concentrations between varieties from contrasting breeding programs (e.g., Aszita and Solstice) were at least partially due to differences in foliar (and especially *Septoria*) disease resistance. This view is supported by the results of a recent UK spring wheat variety trial in the UK, which reported that Zebra, the variety which had substantially higher yellow rust severity, had significantly higher grain Cd concentrations than the other five varieties included in the study [57] (Table 5). 

Different to mineral micronutrients, the concentration of phenolic acids in wheat grain was recently reported to be higher in modern than historical wheat varieties and to have increased over time [116]. In addition, when the performance of contrasting spelt wheat was compared in organic and conventional management systems in a semi-arid region with low foliar disease pressure, grain from the short straw modern spelt variety developed for the conventional sector (Filderstolz) had higher grain phenolic concentrations compared with three long straw varieties developed for the organic farming sector [38]. The higher phenolic concentrations in longer-straw, common wheat varieties developed for the organic sector such as Aszita (Table 1) are therefore unlikely to be linked to straw length. 

However, higher phenolic concentrations in leaves and grain may be explained by selection based on low foliar disease severity in organic breeding programs as synthetic fungicides are prohibited. This hypothesis is supported by the finding that concentrations of phenolic compounds (which can have antimicrobial activity and are part of the plants’ defense mechanism against foliar diseases) are negatively associated with foliar disease severity in wheat [6]. Selection of progeny in organic instead of conventional agronomic backgrounds may also have contributed to differences in phenolic levels between varieties from organic and conventional farming focused breeding programs. This hypothesis is supported by the findings that there are significant differences in (i) pathogen profiles contributing to foliar disease between organic and conventional wheat crops [4,6,7] and (ii) phenolic acid and flavonoid profiles between varieties from organic and conventional winter wheat breeding programs [6]. Most importantly, organic management was reported to result in (i) lower severity disease from obligate pathogens such as powdery mildew and rusts, (ii) similar or slightly lower disease severity from opportunistic pathogens such as *Septoria* and/or (iii) higher levels of disease from seed-borne diseases such as bunt [4,6,7,56]. However, additional studies are required to test these hypotheses and determine to what extent selection for foliar disease severity in organic production backgrounds may co-select for higher grain phenolic and antioxidant concentrations in wheat. 

Recent wheat flour surveys demonstrated that the effect of refining has a substantially larger effect on the concentrations of nutritionally desirable phenolics/antioxidants and mineral micronutrients in wheat flour than primary production systems (organic versus conventional) or wheat genetics (*T. aestivum* vs. *T. spelta*) [72]. Specifically, wholegrain flour had 2–5 times higher antioxidant and mineral levels than white flour and for most parameters assessed significantly higher concentrations in organic compared with conventional wheat flour were only detected when wholegrain brands were compared. However, wholegrain products are estimated to account for less than 20% of total grain consumption in the US and many other countries [117] and conventional wheat breeding over the last 50 years has focused primarily on improving the processing quality of refined grain and/or flour products, especially rapid fermentation-based, white bread production processes [57]. 

In contrast, the organic wheat breeding effort has focused more on improving processing quality parameters, including suitability for stone milling, whole grain flour and slow fermentation/sourdough-based bread making processes, mainly because awareness about the benefits of wholegrain consumption and demand for wholegrain products with high sensory and nutritional quality is thought to be higher and rapidly increasing among organic consumers [57,63]. However, most early stage physiological/biochemical markers (e.g., crude protein, Hagberg Falling Number—an indicator of α-amylase activity, specific weight and protein/gluten profiles) were calibrated against the quality of wheat products made from conventionally produced, refined grain/white flour and are therefore thought to be of limited use for the selection of organic varieties suitable for processing into wholegrain products [32,57,64]. The testing protocols developed/used (by organizations such as the Breadlab at Washington State University, USA) to assess whole grain and long-fermentation/sourdough bread-making quality therefore have little in common with those testing protocols used in short fermentation large-scale commercial baking (e.g., the Chorleywood Process). Specifically, formal tests include speed and tolerance of fermentation, water absorption/retention, loaf volume and crumb appearance, crust appearance, texture and taste and overall flavor and nutritional quality parameters (e.g., fiber and micronutrient concentration), which are also often included in testing protocols for wholegrain-based sourdough bread making quality. 

Testing at early generations is often used because organic growers and processors not only tolerate variation, but often demand it and this speeds up the selection process when the design of population as opposed to pure lines is the target (see also Section 4.4 Evolutionary plant breeding). 

## 5. Conclusions

It is now widely accepted that different trait combinations/phenotypes/resistance profiles are needed for optimum performance of wheat and other cereals in organic and conventional farming systems. This realization has resulted in specialist wheat breeding/selection programs for the organic farming sector in some countries (e.g., the value for cultivation or use (VCU) testing in Germany, Austria and Switzerland). Varieties selected in these programs are now widely used by commercial growers.

Variety comparisons suggest that varieties from organic breeding programs and/or older/traditional wheat varieties used by organic farmers tend to have slightly lower yields, longer straw, greater foliar disease resistance and higher processing and nutritional quality when compared to modern varieties from conventional breeding programs in organic production environments. In contrast, modern short straw, conventional varieties have greater lodging resistance and higher yields when grown in conventional farming backgrounds with intensive use of mineral NPK fertilizers and synthetic pesticides/growth regulators. However, there is still relatively limited published information on the relative performance of varieties from organic and conventional breeding programs in organic farming systems.

There appear to be some synergies between selection for certain agronomic traits in organic breeding programs (e.g., longer straw length and foliar disease resistance against *Septoria* and *Fusarium*) and both processing and nutritional quality traits, but this hypothesis needs to be confirmed in future studies.

Recent studies and reports from Northern Europe also suggest that combining the use of varieties from organic breeding programs with innovative agronomic protocols (e.g., the use of *Rhizobium* inocula and applications of green-waste compost in grass/clover leys grown before cereals to optimize N supply) can; (i) narrow the yield gap between organic and conventional production systems and/or (ii) substantially increase grain protein content and other processing quality parameters in organic wheat production [57,82,118]. This suggests that, different to intensive conventional cereal production, where yields in many European countries have plateaued since the mid-1990s, the productivity of organic production systems is still increasing in Europe. 

There is also a realization that future wheat breeding programs will have to consider the changes in climatic background conditions predicted as a result of global warming [1]. For example, given the evidence that there will be more variable/extreme weather conditions in the future, there is thought to be a need to shift from targeting further increases to maximize yield potential to a focus on traits that increase robustness and yield stability (e.g., resource use efficiency, pest and disease resistance and tolerance to drought and/or waterlogging conditions). However, the design of future wheat breeding/selection programs and EPs should also consider the (a) genetic/physiological differences between spring and winter wheat cultivars and (b) contrasting effects of global warming on the climatic background conditions for spring and winter wheat crops. 

The trend to consider nutritional quality and wholegrain/sourdough bread-making quality parameters in (i) the selection of new varieties or (ii) the design of heterogenous populations is likely to continue, since awareness of the nutritional benefits of wholegrain and long fermentation bread-making processes by organic consumers is likely to increase further in the future. 

Although significant progress can be achieved via improved breeding and selection methods, processors will also have to focus on innovation. This is of particular importance in regions with maritime/temperate climates, where it is often difficult for both organic and conventional farmers to achieve the concentrations and quality of protein specified by millers and bakers [4,6,9,48,57,58,74,82,119]. Conventional producers in these regions address this challenge by applying mineral N fertilizer at critical stages of crop growth and organic farmers have tried to mimic this approach by applying permitted organic fertilizers with a high water-soluble, readily plant available N content (e.g., cattle manure slurry or chicken pellets) to established crops [4,7,9,57]. However, this approach may not always work. For example, lack of rainfall at critical growth stages is known to result in insufficient nitrogen uptake, while high rainfall will lead to substantial nitrogen losses and negative environmental impacts [119,120]. This and the rapidly increasing cost of N fertilizers make it increasingly difficult for farmers to achieve an economic gain from late N fertilizer inputs [90,91,92]. In addition, there is now mounting evidence for negative correlation between (i) N fertilizer inputs and concentrations of nutritionally desirable (poly)phenolic/antioxidant and both lodging and disease resistance and (ii) grain yield and protein content in wheat. This makes it increasingly difficult to justify the use of high N fertilizer inputs and/or varieties with a genetic potential for high grain protein levels, given the challenge to maintain both food quality and security for a growing world population. 

As a result, there is also an urgent need to develop new baking protocols which allow the production of high-quality bread and other bakery products from flour with lower protein/gluten concentrations and/or quality. A recent article by Tucker from Camden-BRI [119] describes that “*many of the technical advances in recent years made in gluten-free bakery technology could find applications in bread making with low protein wheat*” and that this may involve “*evaluating possible hybrid technologies, such as lamination and layering, reflecting the needs for dough with different gluten levels at different parts of the process*”. However, they advise against using increased salt levels to achieve an acceptable crumb structure in bread made from flour with low levels of gluten-forming proteins, since there is a need to reduce dietary salt intake. 

## Figures and Tables

**Table 1:** Main effect means ± SE and ANOVA results (*p*-values) for the effects of fertilizer type, fertilizer input level, and crop protection and variety on grain yield, stem lodging, *Septoria* severity, grain protein concentration and leaf phenolic concentrations in winter wheat (*T. aestivum*) in field trials carried out at Nafferton Farm, Northumberland, UK. Results shown are from a re-analysis of previously published data from two growing seasons (October 2009; November 2010) [6].

	Grain	Stem	*Septoria* Severity		Grain Protein	Leaf Phenolics	
	Yield	Lodging	(AUDPC ^1^)		Concentration	Phenolic	Flavonoids
**Factor**	(t/ha)	% **^2^**	Flag leaf	Leaf 2	(%)	**acids** (mg/g)	(mg/g)
**Fertilizer type**							
Cattle manure	2.9 ± 0.14	7 ± 2	187 ± 16	246 ± 23	10.1 ± 0.2	16.6 ± 1.0	13.8 ± 1.2
Mineral N	4.2 ± 0.23	35 ± 6	257 ± 16	272 ± 23	11.4 ± 0.2	11.9 ± 0.7	10.7 ± 1.0
**Fertilizer level**							
170 kg N/ha	3.8 ± 0.24	25 ± 5	218 ± 18	257 ± 23	11.1 ± 0.2	14.1 ± 0.9	11.9 ± 1.1
85 kg N/ha	3.3 ± 0.16	16 ± 4	226 ± 15	261 ± 22	10.4 ± 0.2	14.5 ± 0.9	12.7 ± 1.2
**Crop Protection**							
Conventional	3.8 ± 0.22	13 ± 4	202 ± 14	252 ± 21	10.8 ± 0.2	13.1 ± 0.8	11.9 ± 1.1
Organic	3.3 ± 0.18	28 ± 5	242 ± 18	266 ± 25	10.8 ± 0.2	15.4 ± 1.0	12.7 ± 1.1
**Variety**							
Aszita (OBP)	3.3 ± 0.16	21 **^2^**	168 ± 13	193 ± 19	12.0 ± 0.2	16.2 ± 1.1	14.3 ± 1.3
Solstice (CBP)	3.8 ± 0.24	-	276 ± 17	325 ± 23	9.5 ± 0.1	12.3 ± 0.6	10.2 ± 0.8
**ANOVA** (*p*-values)							
**Main Effects**							
Fertilizer type (FT)	0.0180	0.0002	0.0347	NS	<0.001	0.0033	0.0034
Fertilizer level (FL)	NS	0.0114	NS	NS	0.0005	NS	NS
Crop protection (CP)	0.0007	<0.001	NS	NS	NS	0.0072	NS
Variety (VR)	<0.001	-	<0.001	<0.001	<0.001	<0.001	<0.001
**Interactions**							
FT × FL	T	0.0150	NS	NS	<0.001	NS	NS
FT × CP	NS	0.0079 **^3^**	NS	0.0005	NS	T	NS
FT × VR	<0.001 **^4^**	-	NS	0.0041	0.0040	T **^7^**	0.0155 **^7^**
FL × VR	T	-	0.0035	NS	NS	NS	NS
CP × VR	NS	-	0.0378 **^5^**	NS	0.0289	NS	NS
FT × FL × VR	NS	-	T	0.0316 **^6^**	0.00186 **^6^**	NS	NS

OBP, variety from an organic farming focused breeding program; CBP, modern short straw variety from a conventional farming focused breeding program; NS, not significant; **^1^**, area under the disease progress curve; **^2^**, data for Aszita only, since no lodging was observed in Solstice; **^3^**, for interaction means see Table 2; **^4^**, for interaction means see Table 3; **^5^** for interaction means see Table 4; **^6^**, for interaction means see Table 5; **^7^**, for interaction means see Table 9.

**Table 2:** Interaction means ± SE for the effects of fertilizer type and crop protection on stem lodging (%) in two winter wheat (*T. aestivum*) field trials carried out at Nafferton Farm, Northumberland, UK.

	Factor 1.	Factor 2. Crop Protection	
**Parameter**	**Fertilizer type**	Conventional	Organic
**Stem lodging**	Cattle Manure	2 ± 1 **a B**	5 ± 2 **a B**
(%)	Mineral N	12 ± 4 **b A**	23 ± 6 **a A**

Means are labelled with the same capital letter within each column and lower-case letter within each row and are not significantly different according to Tukey’s honest significant difference test (THSD) test (*p* < 0.05).

**Table 3:** Interaction means ± SE for the effects of fertilizer type and variety on grain yield in two winter wheat (*T. aestivum*) field trials carried out at Nafferton Farm, Northumberland, UK.

	Factor 1	Factor 2. Variety	
**Parameter**	**Fertilizer type**	Aszita (OBP)	Solstice (CBP)
**Grain yield**	Cattle Manure	2.9 ± 0.2 **a B**	2.9 ± 0.2 **a B**
(t/ha)	Mineral N	3.7 ± 0.2 **b A**	4.7 ± 0.4 **a A**

For each parameter, means labelled with the same capital letter within each column and the same lower-case letter within each row are not significantly different according to the THSD test (*p* < 0.05).

**Table 4:** Interaction means ± SE for the effects of crop protection and variety on *Septoria* severity in two winter wheat (*T. aestivum*) field trials carried out at Nafferton Farm, Northumberland, UK.

	Factor 1	Factor 2. Variety choice	
**Parameter**	**Crop Protection**	Aszita (OBP)	Solstice (CBP)
***Septoria* severity**	Conventional	162 ± 18 **b A**	240 ± 20 **a B**
**Flag leaf** (AUDPC **^1^**)	Organic	173 ± 19 **b A**	310 ± 26 **a A**

**^1^**, Area under the disease progress curve. For each parameter, means are labelled with the same capital letter within each column and the same lower-case letter within each row are not significantly different according to the THSD test (*p* < 0.05).

**Table 5:** Interaction means ± SE **^1^** for the effects of fertilizer type, fertilizer input level and variety on *Septoria* severity and grain protein concentrations in two winter wheat (*T. aestivum*) field trials carried out at Nafferton Farm, Northumberland, UK.

	Factor 1	Factor 2	Factor 3. Variety	
Parameter	Fertilizer Type	Fertilizer Level	Aszita (OBP)	Solstice (CBP)
***Septoria* severity**	Cattle Manure	170 kg N/ha	234 ± 42 **A a**	233 ± 44 **B a**
**Leaf 2** (AUDPC **^1^**)		85 kg N/ha	200 ± 52 **A b**	318 ± 47 **A a**
	Mineral N	170 kg N/ha	155 ± 25 **A b**	407 ± 51 **A a**
		85 kg N/ha	184 ± 33 **A b**	341 ± 38 **A a**
**Grain protein**	Cattle Manure	170 kg N/ha	11.1 ± 0.4 **C a**	9.1 ± 0.1 **B b**
**concentration (%)**		85 kg N/ha	11.5 ± 0.3 **BC a**	8.9 ± 0.2 **B b**
	Mineral N	170 kg N/ha	13.7 ± 0.2 **A a**	10.6 ± 0.2 **A b**
		85 kg N/ha	11.9 ± 0.2 **B a**	9.4 ± 0.2 **B b**

**^1^**, Area under the disease progress curve. For each parameter, means are labelled with the same capital letter within the same treatment column; means are labelled with the same lower-case letter in each row and are not significantly different according to the THSD test (*p* < 0.05).

**Table 6:** Main effect means ± SE and ANOVA results (*p*-values) for the effects of site (Courtyard farm, Norfolk, UK; Gilchester farm, Northumberland, UK; Sheepdrove farm, Berkshire, UK) and variety on grain yield, leaf chlorophyll levels (SPAD), and *Septoria* and yellow rust disease severity in spring wheat (*T. aestivum*) grown under organic management regimes. Results shown are from a re-analysis of data previously published by Wilkinson [57].

	Grain	Chlorophyll	*Septoria* Severity ^1^		Yellow Rust Severity ^1^	
	Yield	Levels ^1^	Flag Leaf	Leaf 2	Flag Leaf	Leaf 2
**Factor**	(t/ha)	(SPAD)	(% **^2^**)	(% **^2^**)	(% **^2^**)	(% **^2^**)
**Site**						
Courtyard	4.4 ± 0.1 **a**	42.0 ± 0.5 **b**	0.2 ± 0.1 **b**	6.1 ± 1.3 **b**	1.7 ± 0.5 **b**	0.3 ± 0.1 **b**
Gilchesters	4.2 ± 0.3 **a**	45.6 ± 1.4 **a**	0.5 ± 0.2 **a**	4.8 ± 1.6 **b**	12.8 ± 3.5 **a**	10.5 ± 3.9 **a**
Sheepdrove	2.9 ± 0.1 **b**	42.3 ± 0.8 **b**	0.2 ± 0.1 **b**	11.1 ± 1.9 **a**	11.1 ± 2.7**a**	9.5 ± 2.6 **a**
**Variety**						
Fasan (100 **^3^**)	4.1 ± 0.3 **b**	41.4 ± 1.1 **c**	0.5 ± 0.2 **ab**	12.2 ± 2.1 **a**	7.9 ± 1.8 **b**	3.0 ± 1.0 **b**
Zebra (98 **^3^**)	2.8 ± 0.4 **e**	37.6 ± 1.9 **d**	0.1 ± 0.1 **b**	10.9 ± 2.8 **a**	37.0 ± 6.2 **a**	33.5 ± 7.5 **a**
Amaretto (92 **^3^**)	3.8 ± 0.3 **cd**	44.7 ± 1.3 **ab**	0.1 ± 0.1 **b**	3.7 ± 0.8 **c**	2.9 ± 0.6 **bc**	1.2 ± 0.3 **b**
Paragon (90 **^3^**)	4.1 ± 0.3 **bc**	46.3 ± 1.0 **a**	0.1 ± 0.1 **b**	4.6 ± 1.0 **b**	0.0 ± 0.0 **c**	0.0 ± 0.0 **b**
Monsun (90 **^3^**)	3.7 ± 0.3 **d**	44.0± 1.0 **b**	0.8 ± 0.3 **a**	11.2 ± 3.8 **a**	3.2 ± 0.7 **bc**	2.9 ± 0.8 **b**
Tybalt (81 **^3^**)	4.6 ± 0.3 **a**	5.9 ± 0.9 **ab**	0.2 ± 0.1 **b**	1.4 ± 0.3 **c**	0.0 ± 0.0 **c**	0.0 ± 0.0 **b**
**ANOVA**						
**Main Effects**						
Site (TS)	<0.001	0.0003	0.0158	0.0083	<0.001	<0.001
Variety (VR)	<0.001	<0.001	<0.001	<0.001	<0.001	<0.001
**Interaction**						
TS × VR	<0.001 **^4^**	<0.001 **^4^**	<0.001	0.0053 **^4^**	<0.001 **^4^**	<0.001

Values shown are means ± SE of data from two growing seasons (2006 and 2007). **^1^**, At Growth Stage 65 (GS65); **^2^**, leaf area covered; **^3^**, mean stem length in cm; **^4^**, for interaction means see Table 8. For the same factor, means labelled with the same letter within each column for each treatment are not significantly different according to the THSD test (*p* < 0.05).

**Table 7:** Main effect means ± SE and ANOVA results (*p*-values) for the effects of site (Courtyard farm, Norfolk, UK; Gilchester farm, Northumberland, UK; Sheepdrove farm, Berkshire, UK) and variety on selected processing and nutritional quality parameters in spring wheat (*T. aestivum*) grown under organic management regimes. Results shown are from a re-analysis of data previously published by Wilkinson [57].

	Bread-Making Quality		Toxic			
	Protein	Protein	Metal	Micronutrient Concentration		
	Content	Quality ^1^	Cd	Ca	Zn	Fe
**Factor**	(%)	(%)	(μg/kg)	(mg/g)	(mg/kg)	(mg/kg)
**Site**						
Courtyard	11.5 ± 0.2 **c**	3.35 ± 0.04	35 ± 3 **b**	0.43 ± 0.01 **b**	26 ± 1 **c**	27 ± 1 **b**
Gilchesters	14.5 ± 0.1 **a**	-	44 ± 2 **a**	0.36 ± 0.02 **c**	33 ± 1 **b**	38 ± 1 **a**
Sheepdrove	14.0 ± 0.2 **b**	3.61 ± 0.04	43 ± 2 **a**	0.49 ± 0.02 **a**	44 ± 1 **a**	34 ± 1 **c**
**Variety** (mean stem length in cm)						
Fasan (100)	13.2 ± 0.3 **c**	3.7 ± 0.1 **a**	37 ± 3 **b**	0.46 ± 0.02 **a**	32 ± 2 **bc**	31 ± 1 **c**
Zebra (98)	14.2 ± 0.4 **a**	3.6 ± 0.1 **b**	50 ± 4 **a**	0.45 ± 0.03 **ab**	37 ± 2 **a**	33 ± 1 **b**
Amaretto (92)	12.9 ± 0.3 **c**	3.5 ± 0.1 **bc**	36 ± 3 **b**	0.44 ± 0.02 **ab**	32 ± 2 **c**	32 ± 1 **b**
Paragon (90)	13.6 ± 0.3 **b**	3.3 ± 0.1 **e**	36 ± 3 **b**	0.45 ± 0.03 **ab**	34 ± 2 **bc**	33 ± 1 **b**
Monsun (90)	12.9 ± 0.4 **c**	3.4 ± 0.1 **cd**	48 ± 3 **a**	0.41 ± 0.02 **b**	35 ± 2 **b**	33 ± 1 **b**
Tybalt (81)	13.0 ± 0.4 **c**	3.4 ± 0.1 **d**	38 ± 4 **b**	0.36 ± 0.01 **c**	34 ± 2 **b**	36 ± 1 **a**
**ANOVA**						
**Main Effects**						
Trial site (TS)	<0.001	<0.001	0.0002	<0.001	<0.001	<0.001
Variety (VR)	<0.001	<0.001	<0.001	<0.001	<0.001	<0.001
**Interactions**						
ST × VR	<0.001 **^2^**	0.0095 **^2^**	0.0008 **^2^**	NS	<0.001 **^2^**	0.0145 **^2^**

Means are from two growing seasons (2006 and 2007). **^1^**, Gluten/protein quality by SE-HPLC [fractions (F3/F4)/F1] was only assessed in two of the sites (Courtyard and Sheepdrove) in both years; **^2^**, for interaction means, see Table 8; NS, not significant. For the same factor, means labelled with the same letter within each column are not significantly different according to the THSD test (*p* < 0.05).

**Table 8:** Interaction means ± SE and ANOVA results (*p*-values) for the effects of site (three contrasting locations in the UK) and variety on grain yield, leaf chlorophyll levels (SPAD), *Septoria* and yellow rust disease severity, grain protein content and quality, and grain Zn and Fe concentrations in spring wheat (*T. aestivum*) grown under organic management regimes.

		Variety					
Parameter	Site	Amaretto	Fasan	Monsun	Paragon	Tybalt	Zebra
**Grain yield**	Courtyard	3.8 ± 0.2 **AB**	4.9 ± 0.3 **A**	4.3 ± 0.2 **A**	4.2 ± 0.3 **B**	5.6 ± 0.3 **A**	3.7 ± 0.4 **A**
(t/ha)	Gilchester	4.4 ± 0.9 **A**	4.5 ± 0.7 **AB**	4.1 ± 0.8 **A**	4.8 ± 0.8 **A**	4.3 ± 0.8 **B**	3.2 ± 0.9 **A**
	Sheepdrove	3.2 ± 0.3 **B**	3.0 ± 0.2 **B**	2.7 ± 0.3 **B**	3.2 ± 0.2 **C**	3.9 ± 0.2 **B**	1.6 ± 0.3 **B**
							
**Chlorophyll**	Courtyard	42 ± 1 **B**	40 ± 1 **A**	42 ± 1 **B**	43 ± 1 **B**	43 ± 1 **B**	42 ± 1 **A**
Levels *	Gilchester	48 ± 3 **A**	43 ± 3 **A**	47 ± 2 **A**	50 ± 2 **A**	49 ± 2 **A**	36 ± 5 **B**
(SPAD)	Sheepdrove	45 ± 2 **AB**	41 ± 1 **A**	43 ± 2 **AB**	46 ± 1 **AB**	45 ± 1 **AB**	34 ± 2 **B**
							
***Septoria***	Courtyard	4 ± 1 **A**	8 ± 3 **B**	4 ± 1 **B**	4 ± 1 **A**	2 ± 1 **A**	14 ± 6 **A**
on leaf L2 *	Gilchester	2 ± 1 **A**	5 ± 1 **B**	16 ± 8 **A**	1 ± 1 **A**	1 ± 1 **A**	3 ± 2 **B**
(% **^1^**)	Sheepdrove	5 ± 2 **A**	23 ± 3 **A**	13 ± 8 **AB**	9 ± 2 **A**	1 ± 0 **A**	15 ± 5 **A**
							
**Yellow rust**	Courtyard	1 ± 1 **A**	1 ± 0 **B**	0 ± 0 **A**	0 ± 0 **A**	0 ± 0 **A**	7 ± 2 **B**
on flag leaf *	Gilchester	4 ± 1 **A**	11 ± 2 **A**	5 ± 2 **A**	0 ± 0 **A**	0 ± 0 **A**	57 ± 18 **A**
(% **^1^**)	Sheepdrove	3 ± 1 **A**	12 ± 4 **A**	4 ± 1 **A**	0 ± 0 **A**	0 ± 0 **A**	47 ± 6 **A**
							
**Protein**	Courtyard	11.5 ± 0.4 **B**	11.2 ± 0.3 **B**	10.6 ± 0.4 **B**	12.3 ± 0.6 **B**	11.0 ± 0.3 **C**	12.3 ± 0.5 **B**
**content**	Gilchester	13.8 ± 0.2 **A**	14.5 ± 0.3 **A**	14.1 ± 0.3 **A**	14.7 ± 0.1 **A**	15.0 ± 0.3 **A**	15.1 ± 0.4 **A**
(%)	Sheepdrove	13.5 ± 0.3 **A**	13.9 ± 0.4 **A**	14.0 ± 0.3 **A**	14.1 ± 0.4 **A**	13.1 ± 0.4 **B**	15.2 ± 0.2 **A**
							
**Protein**	Courtyard	3.4 ± 0.1 **B**	3.6 ± 0.1 **B**	3.2 ± 0.1 **B**	3.2 ± 0.1 **A**	3.3 ± 0.1 **B**	3.4 ± 0.1 **B**
Quality *	Gilchester	-	-	-	-	-	-
(%)	Sheepdrove	3.6 ± 0.1 **A bc**	3.9 ± 0.1 **A a**	3.7± 0.1 **A b**	3.3 ± 0.1 **A d**	3.5 ± 0.1 **A c**	3.7 ± 0.1 **A b**
							
**Grain Zn**	Courtyard	24 ± 1 **C**	24 ± 1 **C**	26 ± 2 **C**	28 ± 1 **C**	24 ± 2 **C**	28 ± 2 **C**
**concentration**	Gilchester	32 ± 1 **B**	32 ± 2 **B**	34 ± 2 **B**	32 ± 1 **B**	36 ± 2 **B**	35 ± 1 **B**
(mg/kg)	Sheepdrove	41 ± 1 **A**	42 ± 2 **A**	46 ± 2 **A**	42 ± 2 **A**	42 ± 2 **A**	48 ± 2 **A**
							
**Grain Fe**	Courtyard	27 ± 2 **C**	25 ± 2 **C**	25 ± 1 **B**	29 ± 2 **C**	29 ± 1 **C**	27 ± 1 **B**
**concentration**	Gilchester	37 ± 2 **A**	36 ± 2 **A**	38 ± 3 **A**	38 ± 2 **A**	42 ± 2 **A**	38 ± 1 **A**
(mg/kg)	Sheepdrove	33 ± 1 **B**	31 ± 1 **B**	35 ± 1 **A**	33 ± 1 **B**	36 ± 1 **B**	35 ± 1 **A**

* At Growth Stage 65 (GS65); **^1^**, leaf area covered. For each parameter, means labelled with the same capital letter within each column and lower-case letter within each row are not significantly different (THSD, *p* < 0.05).

**Table 9:** Interaction means ± SE for the effects of fertilizer type and variety on leaf phenolic acid and flavonoid and grain Cd concentrations in two winter wheat (*T. aestivum*) field trials carried out at Nafferton Farm, Northumberland, UK.

	Factor 1	Factor 2. Variety	
Parameter	Fertilizer Type	Aszita (OBP)	Solstice (CBP)
Leaf phenolic acid	Cattle Manure	19 ± 2 **a**	14 ± 1 **b**
concentrations (mg/g)	Mineral N	12 ± 2 **bc**	11 ± 1 **c**
			
Leaf flavonoid	Cattle Manure	17 ± 2 **a**	11 ± 1 **bc**
concentrations (mg/g)	Mineral N	12 ± 2 **b**	9 ± 1 **c**
			
Grain Cd	Cattle Manure	9.9 ± 0.9 **b**	8.3 ± 0.8 **b**
concentrations (µg/kg)	Mineral N	16.3 ± 1.5 **a**	10.7 ± 0.7 **b**

For each parameter, means labelled with the same letter are not significantly different according to the THSD test (*p* < 0.05).

## Data Availability

The datasets generated and/or re-analyzed for the results represented in the tables are available from the author Leonidas Rempelos upon reasonable request.

## Supplementary Materials

The following supporting information can be downloaded at: https://www.mdpi.com/article/10.3390/foods12061209/s1, Results and discussion of Supplementary correlation analyses; Supplementary Figure S1. Spearman Rank correlations between (i) disease severity (*Septoria* on the flag leaf, SL1; *Septoria* on the 2nd leaf, SL2; yellow rust on the flag leaf, YRL1; yellow rust on the 2nd leaf, YRL2), (ii) crop performance parameters (leaf chlorophyll levels, SPAD; stem length, S.LEN; grain yield, YIELD; thousand grain weight, TGW; protein content, PRO; protein quality, PQ; Hagberg Falling Number, HFN), (iii) grain mineral concentrations (calcium, Ca; iron, Fe; Zinc, Zn) and concentrations of the toxic metal cadmium (Cd) identified in a re-analysis of previously published data from variety trials comparing six contrasting spring wheat varieties in two growing seasons on three organic farms located in contrasting pedo-climatic zones of the UK [57].
