# Supplementary material for: Breeding Bread-Making Wheat Varieties for Organic Farming Systems: The Need to Target Productivity, Robustness, Resource Use Efficiency and Grain Quality Traits
Source: Foods. 2023 Mar 13;12(6):1209. doi: 10.3390/foods12061209 (PMC10048768; doi:10.3390/foods12061209)
Supplement: Supplementary file 1 [file foods-12-01209-s001.zip › foods-2130063-supplementary.pdf]

*Supplementary Material for Review*

# Breeding Bread-Making Wheat Varieties for Organic Farming Systems: The Need to Target Productivity, Robustness, Resource Use Efficiency and Grain Quality Traits

**Leonidas Rempelos [1,2], Juan Wang [3], Enas Khalid Sufar [2], Mohammed Saleh Bady Almuayrifi [2,4], Daryl Knutt [2], Halima Leifert [2], Alice Leifert [2], Andrew Wilkinson [2,5,*], Peter Shotton [2], Gultekin Hasanaliyeva [2,6], Paul Bilsborrow [2], Steve Wilcockson [2], Nikolaos Volakakis [2,7], Emilia Markellou [8], Bingqiang Zhao [9], Stephen Jones [10], Per Ole Iversen [11,12] and Carlo Leifert [11,13,*]**

1 Lincoln Institute for Agri-Food Technology, University of Lincoln, Lincoln LN2 2LG, UK
2 Nafferton Ecological Farming Group, Newcastle University, Newcastle upon Tyne NE1 7RU, UK
3 School of Agriculture and Biology, Shanghai Jiao Tong University, Shanghai 200240, China
4 Almadinah Regional Municipality, Medina 2020, Saudi Arabia
5 Gilchester Organics, Stamfordham NE18 0QL, UK
6 School of Animal, Rural and Environmental Sciences, Nottingham Trent University, Brackenhurst Campus, Nottinghamshire NG25 0QF, UK
7 Geokomi Plc, Sivas Festos, GR70200 Crete, Greece
8 Benaki Phytopathological Institute, 14561 Athens, Greece
9 Institute of Agricultural Resources and Regional Planning (IARRP), Chinese Academy of Agricultural Science (CAAS), Beijing 100081, China
10 Bread Lab, Department of Crop and Soil Sciences, Washington State University, Burlington, WA 98233, USA
11 Department of Nutrition, Institute of Basic Medical Sciences (IMB), University of Oslo, 0317 Oslo, Norway
12 Department of Haematology, Oslo University Hospital, 0372 Oslo, Norway
13 SCU Plant Science, Southern Cross University, Military Rd., Lismore 2480, Australia
* Correspondence: andrew@gilchester.co.uk (A.W.); carlo.leifert@scu.edu.au (C.L.)

## Supplementary correlation analyses

Correlation analyses were carried out to investigate associations between crop health, productivity and grain processing and nutritional quality parameters in organic farming background conditions. Analyses use data from a unique program of variety trials which compared six contrasting spring wheat (*T. aestivum*) in two growing seasons on three organic farms located in contrasting pedo-climatic zones of the UK (see Tables 6-8 in the review article for results of the ANOVAs carried out using the same dataset) [57]. Spearman's correlations were performed by using the 'cor' function and visualized by using the 'corrplot' package in R (www.r-project.org).

*Correlations between disease severity, crop performance and grain processing/nutritional quality parameters*

There were strong positive correlations between the two foliar diseases (yellow rust and *Septoria*) (Supplementary Figure 1), which may indicate that the two diseases are controlled by similar resistance mechanisms in the two varieties (Paragon and Tybald), which had very low disease severity.

Stem length was found to be positively correlated with both diseases, although the correlation coefficient was higher for yellow rust (Supplementary Figure 1). For Septoria, this contrasts with the results obtained in a study of winter wheat performance in organic production systems, where a longer straw variety (Aszita) from an organic breeding program had lower *Septoria* severity compared with a modern short straw variety developed for the conventional farming sector [6]. However, it should be pointed out that *Septoria* severity was relatively low in all spring wheat varieties included in the trials, when compared to studies with winter wheat in an organic farming background in the UK [4,6].

As expected, the severity of both diseases was negatively correlated with leaf chlorophyll levels and grain yield, but there were no correlations with protein content and TGW and weak positive correlations with protein quality

(Figure Table 1). However, the HFN was also positively correlated with yellow rust severity, and there was a weak negative correlation between HFN and *Septoria* severity (Supplementary Figure 1).

Both yellow rust and *Septoria* severity were positively correlated with grain Ca and Cd concentrations, although correlation coefficients were lower for Cd (Figure Table 1). In contrast, there was no correlation between disease severity and grain Zn concentration and negative correlations between both diseases and grain Fe concentrations (Figure Table 1).

Although it is not possible to extrapolate causal relationships between disease severity and crop performance and grain processing/nutritional quality parameters, overall, the correlation analyses suggest that there is a need to breed/select for rust and *Septoria* resistance in breeding programs for the organic sector (which prohibits the use of synthetic fungicides for foliar disease management). Breeding for increased disease resistance has the potential to not only increase grain yield/yield stability, but also to improve some nutritional quality parameters (e.g., reduce grain Cd and increase grain Fe concentrations) (Figure Table 1). In this context, the strong negative correlation between yield and grain Cd concentrations is of particular importance, since cereals are a major dietary source for this toxic metal [1,35,71,111].

*Correlations between crop physiological/performance and grain processing/nutritional quality parameters*

The crop physiological/performance parameters assessed were (i) leaf chlorophyll levels (SPAD), (ii) stem length and (iii) grain yield. When correlations between these three crop physiological/performance parameters were assessed, a strong positive correlation between leaf chlorophyll content and yield, but weak negative correlation between stem length and both (i) leaf chlorophyll and (ii) yield, were detected (Supplementary Figure 1).

Both leaf chlorophyll and grain yield were negatively correlated with grain protein content and quality. However, there was a weak positive correlation between stem length and protein content, but a weak negative correlation with protein quality (Supplementary Figure 1).

Leaf chlorophyll was also negatively correlated with HFN, but there was no correlation with TGW. In addition, there was no correlation between yield and TGW, but there was a weak positive correlation between yield and TGW (Supplementary Figure 1). In contrast, stem length was strongly positively correlated with HFN but negatively correlated with TGW.

Leaf chlorophyll content was negatively correlated with grain Ca and Cd, but there were weak positive correlations between leaf chlorophyll content and both grain Fe and Zn concentrations (Supplementary Figure 1). Grain yield was positively correlated with grain Ca, but was negatively correlated with grain Cd, Fe and Zn concentration, with the highest correlation coefficient being found for Cd (Supplementary Figure 1). Stem length was strongly positively correlated with grain Ca, but there were weak negative correlations between stem length and grain Cd, Fe and Zn concentrations (Supplementary Table 1).

Although it is not possible to extrapolate causal relationships between crop physiological/performance and grain processing/nutritional quality parameters, the negative correlations between grain yield, and both (i) protein content and quality and (ii) grain Cd, Fe and Zn concentrations, suggest that there may be a dilution effect when varieties with a higher yield potential are used in organic farming systems, as previously reported for conventional systems. Similarly, the finding of positive correlations between stem length and (i) HFN, (ii) protein content and (iii) TGW is consistent with previous studies that reported (i) negative correlations between grain yield and protein content and (ii) positive correlations between the stem length of wheat varieties and protein contents and/or bread-making quality of wheat grain in both organic and conventional farming systems [1,6,32,33,37,38,57,74,82].

It is, however, more difficult to explain the correlations between leaf chlorophyll levels and grain processing/nutritional quality parameters. This is mainly because foliar diseases are known to have a negative effect on leaf chlorophyll levels and the negative correlations between chlorophyll levels and both *Septoria* and yellow rust severity (Supplementary Figure 1) suggest that this was also the case in the variety trials reported here. It therefore remains unclear whether and to what extent the negative correlations between chlorophyll levels and Ca, Cd, HFN and protein content and quality were linked to genetic differences in chlorophyll levels and/or foliar disease resistance between the varieties included in the study.

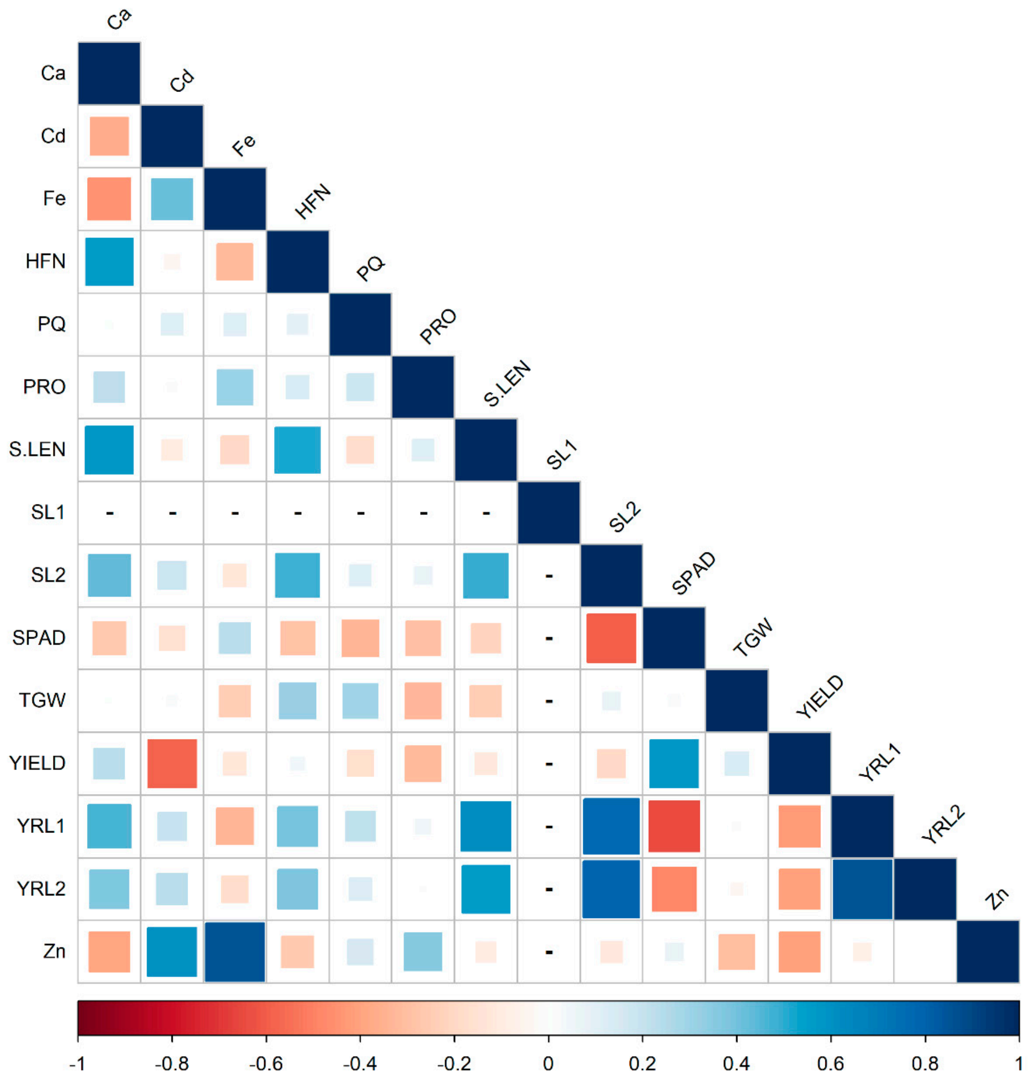


**Supplementary Figure S1.** Spearman Rank correlations between (i) disease severity (*Septoria* on the flag leaf, SL1; *Septoria* on the 2nd leaf, SL2; yellow rust on the flag leaf, YRL1; yellow rust on the 2nd leaf, YRL2), (ii) crop performance parameters (leaf chlorophyll levels, SPAD; stem length, S.LEN; grain yield, YIELD; thousand grain weight, TGW; protein content, PRO; protein quality, PQ; Hagberg Falling Number, HFN), (iii) grain mineral concentrations (calcium, Ca; iron, Fe; Zinc, Zn) and concentrations of the toxic metal cadmium (Cd) identified in a re-analysis of previously published data from variety trials comparing six contrasting spring wheat varieties in two growing seasons on three organic farms located in contrasting pedo-climatic zones of the UK [57].

Correlation coefficients are shown in the graph as color shades with blue colors representing positive correlations and red colors representing negative correlations. Strong correlations are shown as large squares with dark colors, while weaker correlations are shown as light colors and smaller squares.
